# Supplementary material for: Preoperative CA125 Significantly Improves Risk Stratification in High-Grade Endometrial Cancer
Source: Cancers (Basel). 2023 May 4;15(9):2605. doi: 10.3390/cancers15092605 (PMC10177432; doi:10.3390/cancers15092605)
Supplement: Supplementary file 1 [file cancers-15-02605-s001.zip › cancers-2282164-supplementary.pdf]

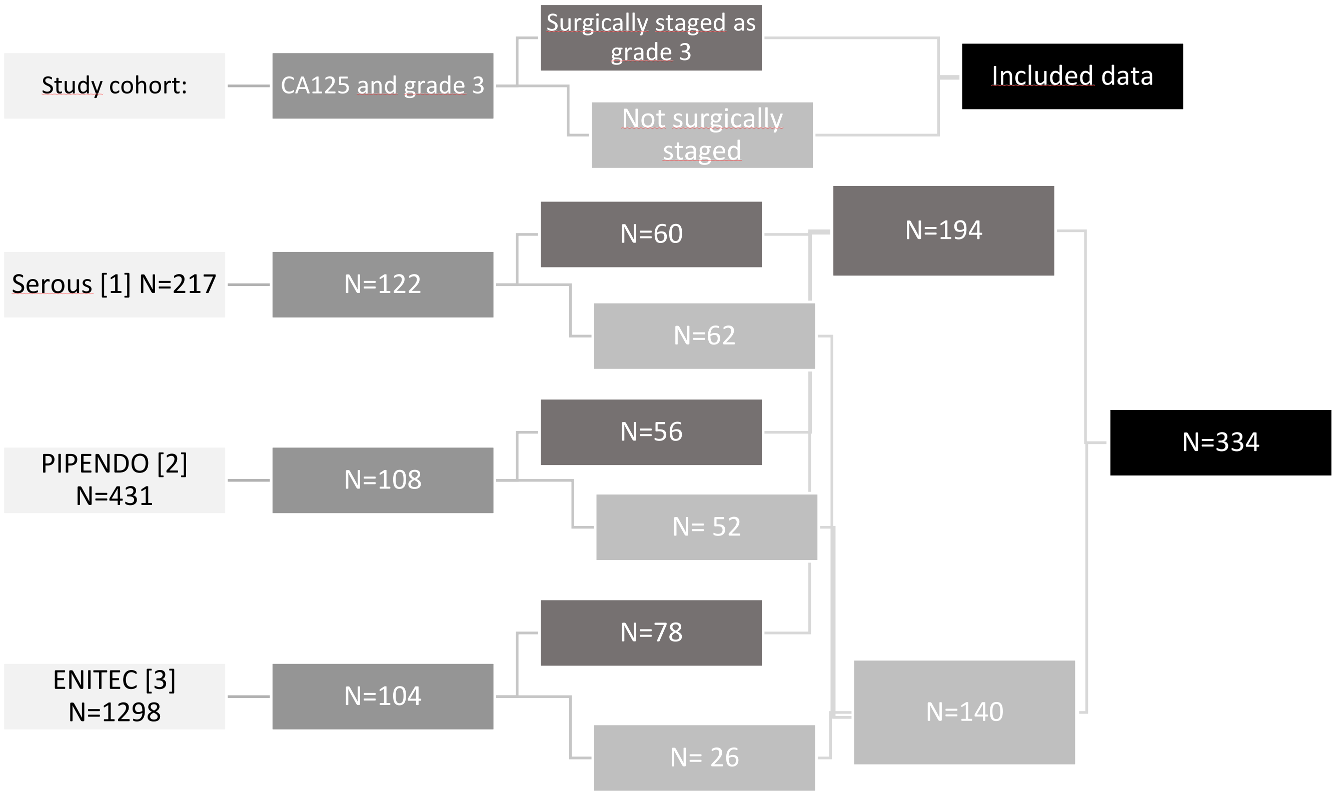

Figure S1. Inclusion of patients

References:

1. Steenbeek MP, Bulten J, Snijders M, Lombaers M, Hendriks J, van den Brand M, et al. Fallopian tube abnormalities in uterine serous carcinoma. Gynecol Oncol. 2020.
2. Visser NCM, van der Wurff AAM, IntHout J, Reijnen C, Dabir PD, Soltani GG, et al. Improving preoperative diagnosis in endometrial cancer using systematic morphological assessment and a small immunohistochemical panel. Hum Pathol. 2021;117:68-78.
3. Reijnen C, Gogou E, Visser NCM, Engerud H, Ramjith J, van der Putten LJM, et al. Preoperative risk stratification in endometrial cancer (ENDORISK) by a Bayesian network model: A development and validation study. PLoS Med. 2020;17(5):e1003111.
